# Adipose-derived stem cells promote glycolysis and peritoneal metastasis via TGF-β1/SMAD3/ANGPTL4 axis in colorectal cancer

**DOI:** 10.1007/s00018-024-05215-1

**Published:** 2024-04-21

**Authors:** Chaojun Zhu, Lan Teng, Yihong Lai, Xingxing Yao, Yuxin Fang, Zihuan Wang, Simin Lin, Haonan Zhang, Qingyuan Li, Ye Li, Jianqun Cai, Yue Zhang, Changjie Wu, Bing Huang, Aimin Li, Side Liu, Qiuhua Lai

**Affiliations:** 1 Guangdong Provincial Key Laboratory of Gastroenterology, Department of Gastroenterology, Nanfang Hospital, Southern Medical University, 1838 Guangzhou Avenue North, Guangzhou, 510515 Guangdong People’s Republic of China; 2 Department of General Surgery and Guangdong Provincial Key Laboratory of Precision Medicine for Gastrointestinal Tumor, Nanfang Hospital, Southern Medical University, Guangzhou, Guangdong China; 3 Department of Plastic and Cosmetic Surgery, Nanfang Hospital, Southern Medical University, Guangzhou, Guangdong China; 4 Department of Gastroenterology, Zhuhai People’s Hospital (Zhuhai Hospital Affiliated with Jinan University), Zhuhai, Guangdong China

**Keywords:** Colorectal carcinoma, Peritoneal carcinomatosis, Adipose-derived mesenchymal stem cells, ANGPTL4, TGF-β/SMAD, Warburg effect

## Abstract

Peritoneal metastasis, the third most common metastasis in colorectal cancer (CRC), has a poor prognosis for the rapid progression and limited therapeutic strategy. However, the molecular characteristics and pathogenesis of CRC peritoneal metastasis are poorly understood. Here, we aimed to elucidate the action and mechanism of adipose-derived stem cells (ADSCs), a prominent component of the peritoneal microenvironment, in CRC peritoneal metastasis formation. Database analysis indicated that ADSCs infiltration was increased in CRC peritoneal metastases, and high expression levels of ADSCs marker genes predicted a poor prognosis. Then we investigated the effect of ADSCs on CRC cells in vitro and in vivo. The results revealed that CRC cells co-cultured with ADSCs exhibited stronger metastatic property and anoikis resistance, and ADSCs boosted the intraperitoneal seeding of CRC cells. Furthermore, RNA sequencing was carried out to identify the key target gene, angiopoietin like 4 (ANGPTL4), which was upregulated in CRC specimens, especially in peritoneal metastases. Mechanistically, TGF-β1 secreted by ADSCs activated SMAD3 in CRC cells, and chromatin immunoprecipitation assay showed that SMAD3 facilitated ANGPTL4 transcription by directly binding to ANGPTL4 promoter. The ANGPTL4 upregulation was essential for ADSCs to promote glycolysis and anoikis resistance in CRC. Importantly, simultaneously targeting TGF-β signaling and ANGPTL4 efficiently reduced intraperitoneal seeding in vivo. In conclusion, this study indicates that tumor-infiltrating ADSCs promote glycolysis and anoikis resistance in CRC cells and ultimately facilitate peritoneal metastasis via the TGF-β1/SMAD3/ANGPTL4 axis. The dual-targeting of TGF-β signaling and ANGPTL4 may be a feasible therapeutic strategy for CRC peritoneal metastasis.

**Supplementary Information:**

The online version contains supplementary material available at 10.1007/s00018-024-05215-1.

## Introduction

Colorectal cancer (CRC), one of the most prevalent malignant tumors with high morbidity and poor prognosis, is the second leading cause of cancer-related death worldwide [1, 2]. Along with hepatic and pulmonary metastasis, intraperitoneal dissemination is one of the most common metastasis in CRC. It becomes a noticeable problem that patients with peritoneal metastasis have a poor prognosis for the rapid progression and limited treatment strategy [3, 4]. Around 4–19% of CRC patients are diagnosed with synchronous or metachronous peritoneal metastases, which are related to significantly shorter overall survival and progression-free survival compared with metastatic CRC in other sites [5, 6]. Therefore, it is urgent to elucidate the mechanisms underlying the process of peritoneal metastasis in CRC and develop potential treatment strategies.

Emerging evidence indicates that adipose-derived stem cells (ADSCs) embedded within the adipose tissues can be recruited into the tumor microenvironment by tumor-released chemokines, especially within an obese state [7–9]. Recent studies indicated that ADSCs in the tumor microenvironment participated in dysregulating vital pathways and influenced progression in multiple cancer [9–12]. The greater omentum, which is abundant of adipose tissues, contains various stem cells and immune cells and endows the reciprocal actions between cancer cells and surrounding cells in the tumor microenvironment [13, 14]. Cancer-related ADSCs secrete plenty of factors, including VEGF, IL-6, TGF-β1, CCL2, and so on [9, 15]. Thereinto, IL-6 and HGF secreted by visceral adipose stromal cells promote vasculogenesis and the formation of hepatic and pulmonary metastasis in CRC [9]. However, whether ADSCs contribute to peritoneal metastasis in CRC remains unclear.

Angiopoietin like 4 (ANGPTL4), an adipokine involved in lipid and glucose metabolism, plays a critical role in cancer development [16]. The mRNA level of ANGPTL4 in colorectal tumor is higher than that in adjacent normal mucosa [17]. Moreover, plasma level of ANGPTL4 markedly declined after tumor resection in CRC patients [18]. ANGPTL4 plays pivotal roles in regulating glucose homeostasis and lipid metabolism, and thereby affects the invasion and chemotherapy resistance of tumor cells [19–21]. In addition, the induction of ANGPTL4 by TGF-β/SMAD signaling was reported to enhance the retention and seeding of breast cancer cells in the lungs [22]. Nevertheless, the regulatory role of TGF-β/SMAD signaling in ANGPTL4 expression has not yet been elucidated clearly.

To explore the contextual role of ADSCs in CRC peritoneal metastasis and the mechanism by which ADSCs may instigate metastasis, we first isolated ADSCs from visceral adipose tissues of CRC patients and investigated their influence on the invasive behavior and anoikis resistance of CRC cells in vitro and in vivo. Next, RNA sequencing analysis was carried out to identify the key target gene and pathway. Furthermore, we investigated the mechanism that ADSCs elevated ANGPTL4 expression via TGF-β1/SMAD3 signaling pathway, thereby promoting glycolysis in CRC cells. Our data suggest that the paracrine effect of ADSCs is essential to CRC peritoneal metastasis and provide insights for novel therapeutic approaches for CRC patients with peritoneal metastasis.

## Materials and methods

### Clinical samples and cell lines

Colorectal cancer specimens and corresponding normal colon tissues for quantitative real-time PCR (qRT-PCR) and western blot analysis were acquired from patients who underwent surgery at the Department of General Surgery, Nanfang Hospital, Southern Medical University. All patients were pathologically diagnosed with CRC and none of them received chemotherapy or radiation therapy before surgery. The colorectal cancer specimens were collected with permission from the Medical Ethics Committee of Nanfang Hospital. All patients were informed and written consent forms.

Human normal colorectal cell line (FHC), and human colorectal cancer cell lines (SW620, HT29, LoVo, SW480, RKO and HCT116) were obtained from the Cell Bank of Type Culture Collection (Chinese Academy of Sciences, Shanghai, China). The cells were cultured in the corresponding medium as recommended, at 37 °C in a humidified atmosphere with 5% CO_2_.

### ADSCs isolation, culture and characterization

ADSCs were isolated from the greater omentum or adipose tissues adjacent to colorectal tumors of CRC patients. Clinical characteristics of CRC patients for the sources of ADSCs are listed in Supplementary Table S1. As previously reported [23], fresh adipose tissues were washed three times with cold PBS containing 1% penicillin–streptomycin and 100 µg/ml gentamicin and minced with sterile scissors. Then, the adipose pieces were digested by solution containing 1.5 mg/ml collagenase type IV (Gibco, Carlsbad, USA) and 20 µg/ml hyaluronidase (Sigma-Aldrich, St Louis, USA) on shaker for 30 min at 37 °C. After incubation, the suspension was then filtered with a 100-µm cell strainer to remove residual tissues and the cells in the filtrate were collected and resuspended in complete medium for human adipose-derived mesenchymal stem cells (HUXMD-90011, Cyagen, Guangzhou, China) with 10% FBS and 1% penicillin–streptomycin. The medium was changed every 2 or 3 days and cells were passaged at approximately 80% confluency. Early passages (second to tenth passages) of ADSCs were utilized for experiments in this study. Ten different ADSCs lines were obtained and used for independent experiments.

The ADSCs were identified by flow cytometry with the Human MSC Analysis Kit (562245, BD Biosciences, San Jose, USA) according to the manufacturer’s instructions. To identify the multi-lineage differentiation potential, ADSCs were cultured in an induction differentiation medium (Cyagen) for adipogenesis, osteogenesis, and chondrogenesis following the manufacturer’s instructions.

### Conditioned medium and co-culture system

To produce conditioned medium (CM) from ADSCs, cells were transferred into a 6-well plate (1 × 10^5^ cells/well) and the culture medium was changed with serum-free medium the next day. After 48 h of incubation, the supernatant was collected and centrifuged at 500*g* for 10 min, following sterilization with a filter. The inserts with 0.4-µm pore polycarbonate membranes (Corning, New York, United States) were used for indirect co-culture system allowing crosstalk between cells in the lower and upper chamber through paracrine factors.

### Patient-derived peritoneal metastasis (PM) cells isolation and culture

Malignant ascites was obtained from CRC patients who were pathologically or clinically diagnosed with peritoneal metastasis. To isolate the tumor cells, the ascites was centrifuged at 2000*g* for 20 min and red blood cells lysis buffer was added into the cell pellet to remove the red blood cells. The purified PM cells were cultured in DMEM/F12 (Gibco) containing 10% FBS and 1% penicillin–streptomycin.

### Patient-derived organoid isolation and culture

Colorectal tumor tissues were cut into 1–3 mm pieces and further digested with solution containing 1.5 mg/ml collagenase type IV and 20 µg/ml hyaluronidase on shaker for 40 min at 37 °C. The suspension was passed sequentially through 100-µm and 40-µm strainer, and then the multicellular fragments on the mesh of the 40-µm strainer were collected. The resulting cell pellets were resuspended in Matrigel (Corning) and 40 µl suspension was pipetted into each well of a pre-warmed 24-well plate to form domes. After solidification, organoid culture medium was added as described in our previous study [24]. In addition, the organoids were co-cultured with ADSCs in a transwell system and/or dosed with 10 µM LY2157299 (HY-13226, MedChemExpress, New Jersey, USA).

### Exosome isolation

The exosome was isolated from ADSCs by ExoQuick-TC™ Exosome Precipitation Solution (EXOTC10A-1, System Biosciences, Palo Alto, USA). Briefly, culture supernatant of ADSCs was collected and centrifuged at 3000*g* for 15 min to remove cell debris, following filtration with Amicon^®^ Ultra-15 Centrifugal Filter Devices (Millipore, Billerica, USA). Subsequently, the ExoQuick-TC was added into enriched supernatant to precipitate the exosome. Western blot was performed to identify the markers of exosome.

### Enzyme-linked immunosorbent assay (ELISA)

The supernatant TGF-β1 in CRC cells, ADSCs and co-culture system was detected with Human TGF-β1 ELISA Kit (EK981-96, MULTI SCIENCES, Hangzhou, China) according to the manufacturer’s instructions.

### Chromatin immunoprecipitation (ChIP) assay

As descripted in previous study [25], Chromatin Immunoprecipitation (ChIP) Assay Kit (P2078, Beyotime, Shanghai, China) was used to perform ChIP assay as the manufacturer’s instructions. In brief, SW480 cells in a 10-cm dish were cross-linked with 1% formaldehyde, then the acquired chromatin fragment was used for immunoprecipitation by SMAD3 antibody (1:100, #9523, CST) with rabbit IgG antibody as the negative control. The immunoprecipitated DNA was purified and tested by ChIP-PCR and qRT-PCR. Enrichment of indicated DNA region by immunoprecipitation was normalized with input using 2^–ΔCt^ method (% input). The primers used are listed in Supplementary Table S2.

### Lactate production assay

Lactate assay kit (A019-2-1, Nanjing Jiancheng Bioengineering Institute, Nanjing, China) was used according to the manufacturer’s instructions. In briefly, 10 µl culture supernatant of cells was utilized as a reaction sample and 10 µl of double-distilled water was used as a negative control. Then working solution and color developer were added in turn. The mixture was placed in a 37 °C water bath for 10 min and the optical density values were measured at a wavelength of 530 nm.

### Animal models and treatments

Female 5-weeks-old BALB/c nude mice were purchased from the Guangdong Medical Laboratory Animal Centre and maintained in the Laboratory Animal Research Center of Nanfang hospital. For the intraperitoneal dissemination model, a total of 1 × 10^6^ SW480 cells were injected alone or co-injected with 1 × 10^6^ ADSCs into the peritoneal cavity of the mice. Five days later, 20 mg/kg LY2157299 or vehicle was injected intraperitoneally every day for 18 days. Tumor growth and intraperitoneal metastasis formation were assessed by an In Vivo Imaging System. Then mice were sacrificed and all the tumors in the abdominal cavity were captured for further analysis. The animal study was approved by the Nanfang Hospital Experimental Animal Ethics Committee.

### Bioinformatic analysis

As described in our previous study [26], The expression profile of 568 colorectal cancer samples and the corresponding clinical data were downloaded from The Cancer Genome Atlas (TCGA) database (https://portal.gdc.cancer.gov/). The expression profile of GSE41568, GSE17536 and GSE161097 were downloaded from the Gene Expression Omnibus (GEO) database (https://www.ncbi.nlm.nih.gov/geo/).

To assess the infiltration of ADSCs in tumor microenvironment, single-sample gene set enrichment analysis (ssGSEA) was applied via GSVA package in R software (version: x64 4.2.1), and the marker genes of ADSCs were downloaded from CellMarker (http://xteam.xbio.top/CellMarker/index.jsp). The survival analysis was perfomed in PROGgeneV2 (http://www.progtools.net/gene/index.php) and GEPIA2 (http://gepia2.cancer-pku.cn/#index). The multivariate Cox regression analysis and forest plots were constructed by survival and survminer package. The volcano map, boxplots, heatmap and bubble charts were constructed using the ggplot2, pheatmap or other packages in R software.

Gene set enrichment analysis (GSEA) was performed using GSEA software (http://www.gsea-msigdb.org/gsea/index.jsp, version 4.2.3). STRING database (http://string-db.org) was used to predict the protein–protein interaction (PPI) network between ANGPTL4 and cytokines produced by mesenchymal stem cells with a combined score > 0.4. JASPAR database (https://jaspar.genereg.net/) was applied to indentify putative SMAD3 binding sites in the ANGPTL4 promoter with a relative score of 80% or over.

### Statistical analysis

GraphPad Prism software (version 8.0, San Diego, USA), IBM SPSS Statistics software (version 22.0, Chicago, USA), and R software (version: x64 4.2.1) were used to perform statistical analysis. Quantitative data are shown as mean ± standard deviation (SD). Statistical significance between two groups was detected by Student’s *t *test. The difference between three or more groups was calculated using One-way ANOVA. The log-rank test was applied for Kaplan–Meier survival analysis. Multivariate Cox regression analysis was performed to estimate hazard ratio (HR) and 95% confidence interval (CI). In all pictures: ns, not significant; *p < 0.05, **p < 0.01, ***p < 0.001, ^###^*p* < 0.001.

Additional methods information can be found in Supplementary Materials.

## Results

### Tumor-infiltrating ADSCs are associated with a poor prognosis in CRC patients

**Fig. 1:**
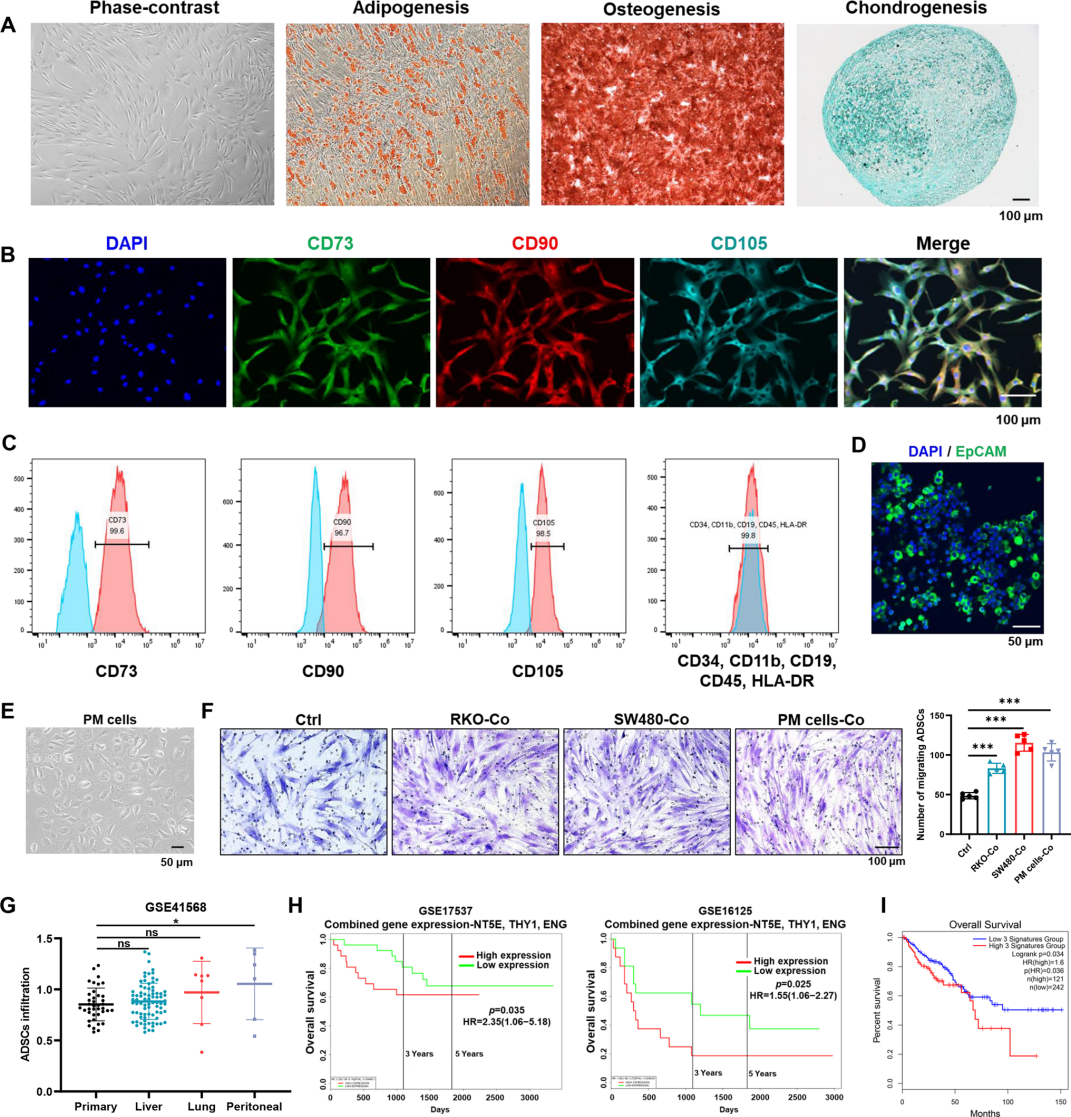
Identification of ADSCs and survival analysis based on ADSCs marker genes. **A** Representative photomicrographs of ADSCs derived from visceral adipose tissues of CRC patients. The multi-lineage differentiation potential was tested by adipogenesis, osteogenesis, and chondrogenesis. Scale bar 100 µm. **B** ADSCs were characterized by positive staining for CD73, CD90 and CD105. Scale bar 100 µm. **C** ADSCs were identified by flow cytometry (blue: isotype control, red: lineage markers). **D** Immunofluorescence staining of EpCAM, an epithelial tumor marker, in ascites cells. *EpCAM* epithelial cell adhesion molecule. Scale bar 50 µm. **E** Third passage PM cells isolated from malignant ascites of CRC patients. Scale bar, 50 µm. **F** Transwell migration assay of ADSCs co-cultured with indicated CRC cells for 48 h. Scale bar 100 µm.** G** The infiltration of ADSCs in primary tumors and liver, lung or peritoneal metastases (data from GSE41568). **H**, **I** Overall survival plots depicting the survival of CRC patients stratified by the combined gene expression levels of NT5E, THY1, and ENG (data from the PROGgeneV2 and GEPIA2 databases). Data are shown as mean ± SD of at least three independent experiments in (D) (Student’s *t *test. ns, not significant; *p < 0.05, ***p < 0.001)

To investigate the effect of ADSCs on the carcinogenesis of CRC, we isolated ADSCs from the greater omentum or adipose tissues adjacent to colorectal tumors of CRC patients. These cells had long shuttle shapes and displayed multi-lineage differentiation potential for adipogenesis, osteogenesis, and chondrogenesis (Fig. 1A). Then, the primary ADSCs were characterized by positive staining for CD73, CD90 and CD105, classical marker genes of mesenchymal stem cell [27], while negative for CD34, CD11b, CD19, CD45 and HLA-DR (Fig. 1B, C). In addition, peritoneal metastasis cells were isolated from malignant ascites of CRC patients, which were membranous stained with EpCAM, an epithelial tumor marker (Fig. 1D, E). To confirm whether ADSCs could be recruited by CRC cells, we performed transwell migration assay. ADSCs exhibited a migration potential that was enhanced by the co-culture with RKO and SW480 cells, as well as PM cells (Fig. 1F). More importantly, ADSCs infiltration increased significantly in CRC peritoneal metastases, but not in liver or lung metastases (Fig. 1G). Furthermore, human clinical data from the PROGgeneV2 and GEPIA2 database revealed that the combined gene expression of NT5E, THY1, and ENG (also known as CD73, CD90 and CD105, respectively) was associated with a poor prognosis (Fig. 1H, I; Supplementary Fig. S1A–F). The above findings implied that ADSCs might play a crucial part in the carcinogenesis of CRC and are associated with a poor prognosis.

### ADSCs promote the metastatic property of CRC cells in vitro and in vivo

**Fig. 2:**
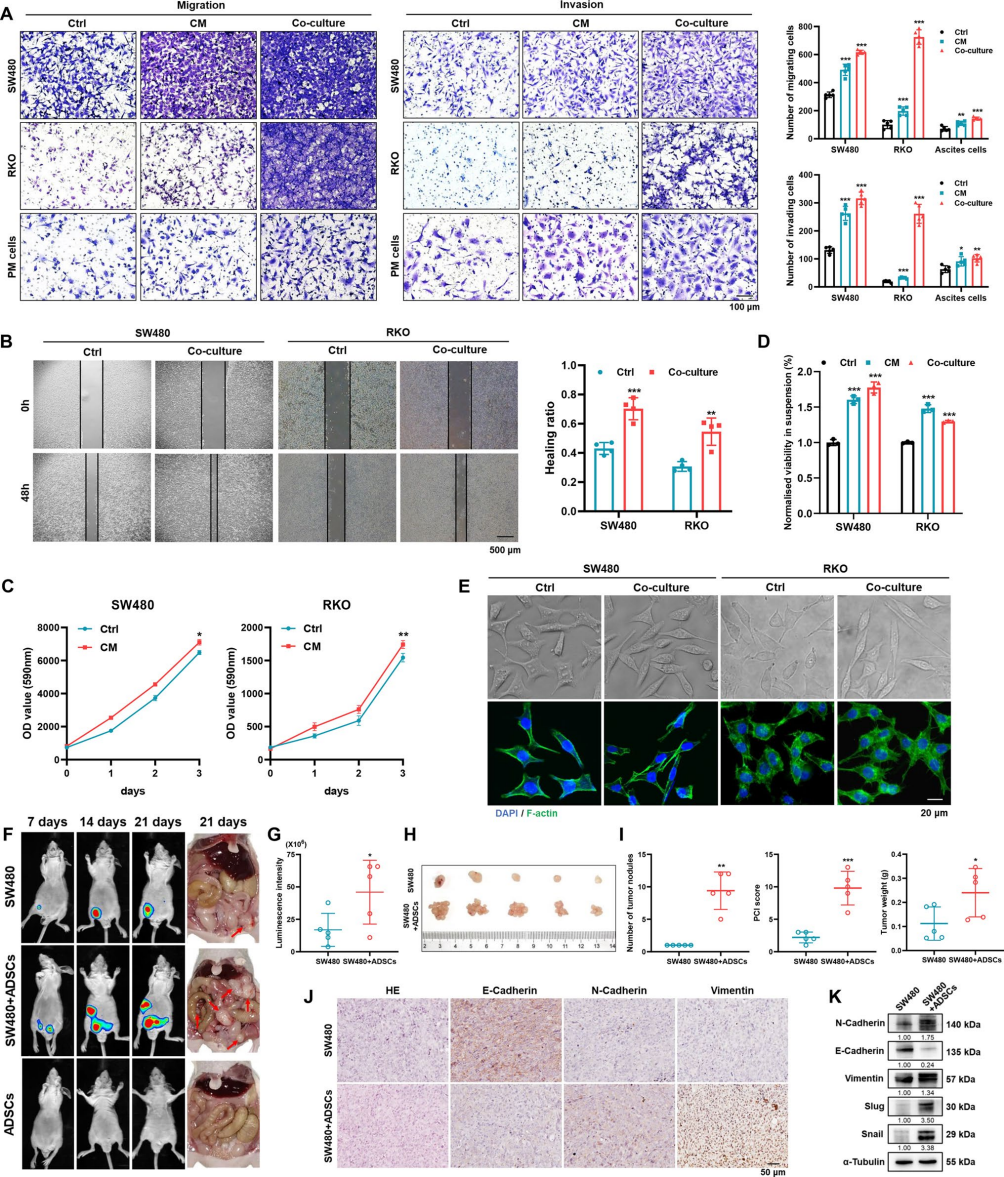
ADSCs enhance the metastatic potential of CRC cells. Cell migration and invasion assay (**A**) and wound healing assay (**B**) in CRC cells treated as indicated. Scale bars 100 µm (**A**) and 500 µm (**B**). **C** Cell proliferation assay on SW480 and RKO cells treated with or without ADSCs-CM. **D** Anoikis assay results indicating that the viability of tumor cells in suspension was substantially enhanced by treatment with ADSCs-CM or ADSCs for 48 h. **E** Phalloidin staining showing the morphologic changes and rearrangement of actin filaments in CRC cells. Scale bar 20 µm. **F**, **G** Whole-body in vivo imaging analysis of mice (n = 5) intraperitoneally injected with cells as indicated to monitor tumor growth and intraperitoneal dissemination. Image of peritoneal metastases (**H**) and analyses for tumor number, weight and PCI score (**I**). IHC staining (**J**) and western blot analysis (**K**) for EMT key molecules in intraperitoneal tumors. Scale bar 50 µm. Data are shown as mean ± SD of at least three independent experiments in (**A**–**D**) (Student’s *t *test. *p < 0.05, **p < 0.01, ***p < 0.001)

We next explored the effect of ADSCs on the metastatic property of CRC cells in vitro. The transwell assay showed that ADSCs-CM and ADSCs significantly increased the number of migrating and invading CRC cells (Fig. 2A). Similarly, co-culture with ADSCs raised the wound healing rate of SW480 and RKO cells from about 40 to 70% and 30–55%, respectively (Fig. 2B). In addition, treatment of ADSCs-CM slightly accelerated the proliferation of SW480 and RKO cells (Fig. 2C). Resistance to anoikis is a critical property in the survival and subsequent colonization to the peritoneum for shed cancer cells in the peritoneal cavity [28], which was determined by measuring the viability of cancer cells in suspension. The result revealed that the anoikis resistance of CRC cells was substantially enhanced by treatment of ADSCs-CM or co-culture with ADSCs (Fig. 2D). Furthermore, using phalloidin staining, we observed that the co-culture with ADSCs mediated the rearrangement of actin filaments, yielding SW480 and RKO cells with an elongated shape and longer filopodia (Fig. 2E).

Then, we investigated whether ADSCs could induce the intraperitoneal dissemination of CRC cells in nude mice. In line with the in vitro results, the intraperitoneal co-injection of ADSCs remarkably enhanced the metastatic ability of CRC cells in the intraperitoneal dissemination model. As the results showed, ADSCs alone did not generate lesions and there was only one tumor nodule per mouse injected with SW480 cells alone, whereas the number of intraperitoneal tumors dramatically increased up to 12 in co-injection group, along with higher peritoneal carcinomatosis index (PCI) score and heavier tumor weight (Fig. 2F–I). Of note, immunohistochemistry (IHC) staining and western blot analysis of xenograft tissues revealed that the co-injection of ADSCs motivated the epithelial-mesenchymal transition (EMT) of SW480 cells in vivo (Fig. 2J, K).

These findings demonstrate that ADSCs boost metastatic property of CRC cells both in vitro and in vivo.

### ADSCs govern the metastatic potential of CRC cells by upregulating ANGPTL4

**Fig. 3:**
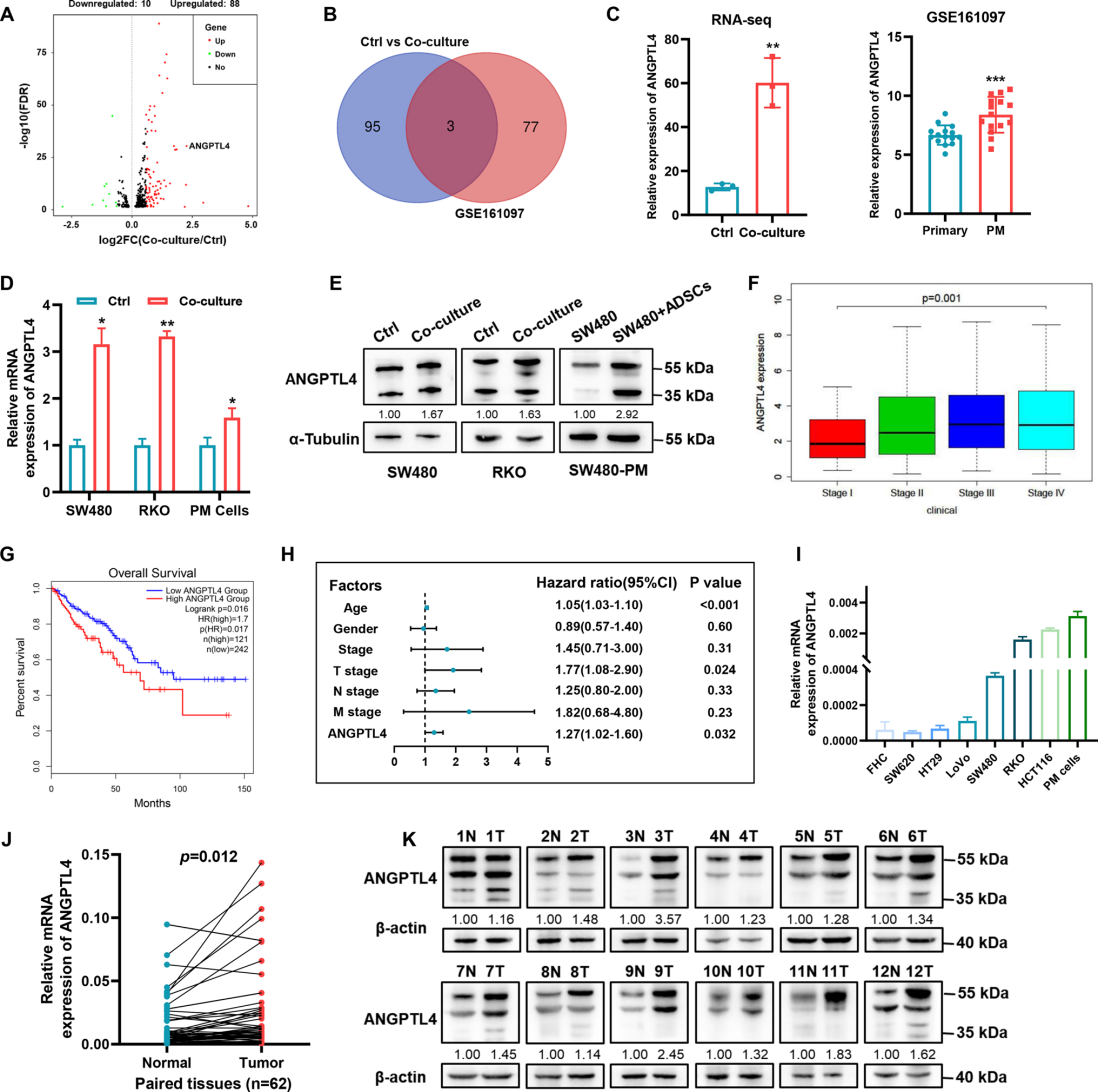
ADSCs facilitate the expression of ANGPTL4. **A** Volcano plot for DEGs in SW480 cells cultured with or without ADSCs. Red: upregulated, green: downregulated, black: not significant; FC: fold change; FDR: false discovery rate. **B** Venn plot showing three overlapping genes among the DEGs identified by RNA sequencing and those in GSE161097. **C** ANGPTL4 expression was strikingly increased in SW480 co-cultured with ADSCs for 48 h (n = 3) and in peritoneal metastases compared with primary tumors (GSE161097, n = 15). **D** qRT-PCR analysis of ANGPTL4 in CRC cells treated as indicated. **E** Western blot analysis of ANGPTL4 in CRC cells and peritoneal metastasis models. **F** ANGPTL4 expression levels over clinical stage progression. **G** Kaplan–Meier of overall survival curve based on ANGPTL4 expression levels (data from GEPIA2). **H** Multivariate Cox regression analysis of clinical features and ANGPTL4 expression levels (data from TCGA-CRC). **I** qRT-PCR analysis for ANGPTL4 mRNA expression in CRC cell lines and PM cells. **J**, **K** Differential expression of ANGPTL4 in fresh colorectal tumor specimens and adjacent normal tissues of patients from our hospital (n = 62 for qRT-PCR and n = 12 for western blot analysis). Data are shown as mean ± SD of at least three independent experiments in (**D**) and (**I**) (Student’s *t *test. *p < 0.05, **p < 0.01, ***p < 0.001). One-way ANOVA for (**F**). Paired-sample *t *test for (**J**)

Given the striking ADSCs-induced promotion in the migration capacity of CRC cells, we then sought to decipher the underlying mechanism. RNA sequencing analysis was conducted to compare the gene expression profiles of SW480 cells cultured with or without ADSCs. A total of 98 differentially expressed genes (DEGs) were obtained with the standard of |log_2_FC|> 0.58 and false discovery rate (FDR) < 0.05. Three of these DEGs were also among the 80 DEGs identified in GSE161097 (Fig. 3A, B; Supplementary Fig. S2A, B), which elucidated the transcriptomic profile of 15 cases of primary colorectal tumors and 15 paired peritoneal metastases [29]. In particular, ANGPTL4, which involved in cancer progression and therapy resistance [19–21], was prominently upregulated both in our RNA sequencing analysis and GSE161097 (Fig. 3C). Consistently, the qRT-PCR analysis indicated the ANGPTL4 mRNA in CRC cell lines was elevated by ADSCs, especially in SW480 and RKO cells (Fig. 3D; Supplementary Fig. S2C). Furthermore, western blot analysis further confirmed that the co-culture with ADSCs significantly upregulated ANGPTL4 expression in SW480 and RKO cells, as well as peritoneal metastases in Fig. 2 (SW480-PM) (Fig. 3E).

Then, we explored the expression profile of ANGPTL4 in TCGA database and observed that the expression of ANGPTL4 increased with the progression of the clinical stages of CRC (Fig. 3F; Supplementary Fig. S2D–F). Notably, data from GEPIA2 indicated that CRC patients with higher ANGPTL4 expression levels had a poorer prognosis than those with lower ANGPTL4 expression levels (Fig. 3G), consistent with the data from GSE29621 and GSE39582 (Supplementary Fig. S2G, H). The multivariate Cox regression analysis on clinical features (age, gender and stage) and ANGPTL4 expression levels revealed that ANGPTL4 could be an independent risk factor for CRC death (TCGA-CRC: HR = 1.27, 95% CI 1.02–1.60, *p* = 0.032; GSE17536: HR = 3.4, 95% CI 1.62–7.2, *p* = 0.001) (Fig. 3H; Supplementary Fig. S2I). In addition, compared with normal colorectal cell line FHC, ANGPTL4 expression levels were significantly increased in CRC cell lines, and PM cells isolated from malignant ascites of CRC patients had much higher expression levels than CRC cell lines (Fig. 3I). Likewise, clinical data from our hospital indicated that ANGPTL4 was upregulated in fresh CRC specimens compared with adjacent normal tissues, confirmed by qRT-PCR (n = 62, p = 0.012) and western blot (n = 12) (Fig. 3J, K).

Taken together, these results demonstrate that ADSCs drive ANGPTL4 mRNA and protein expression in CRC cells, and high ANGPTL4 expression levels are associated with a poor prognosis in patients with CRC.

### ANGPTL4 promotes migration capacity and anoikis resistance in CRC cells

**Fig. 4:**
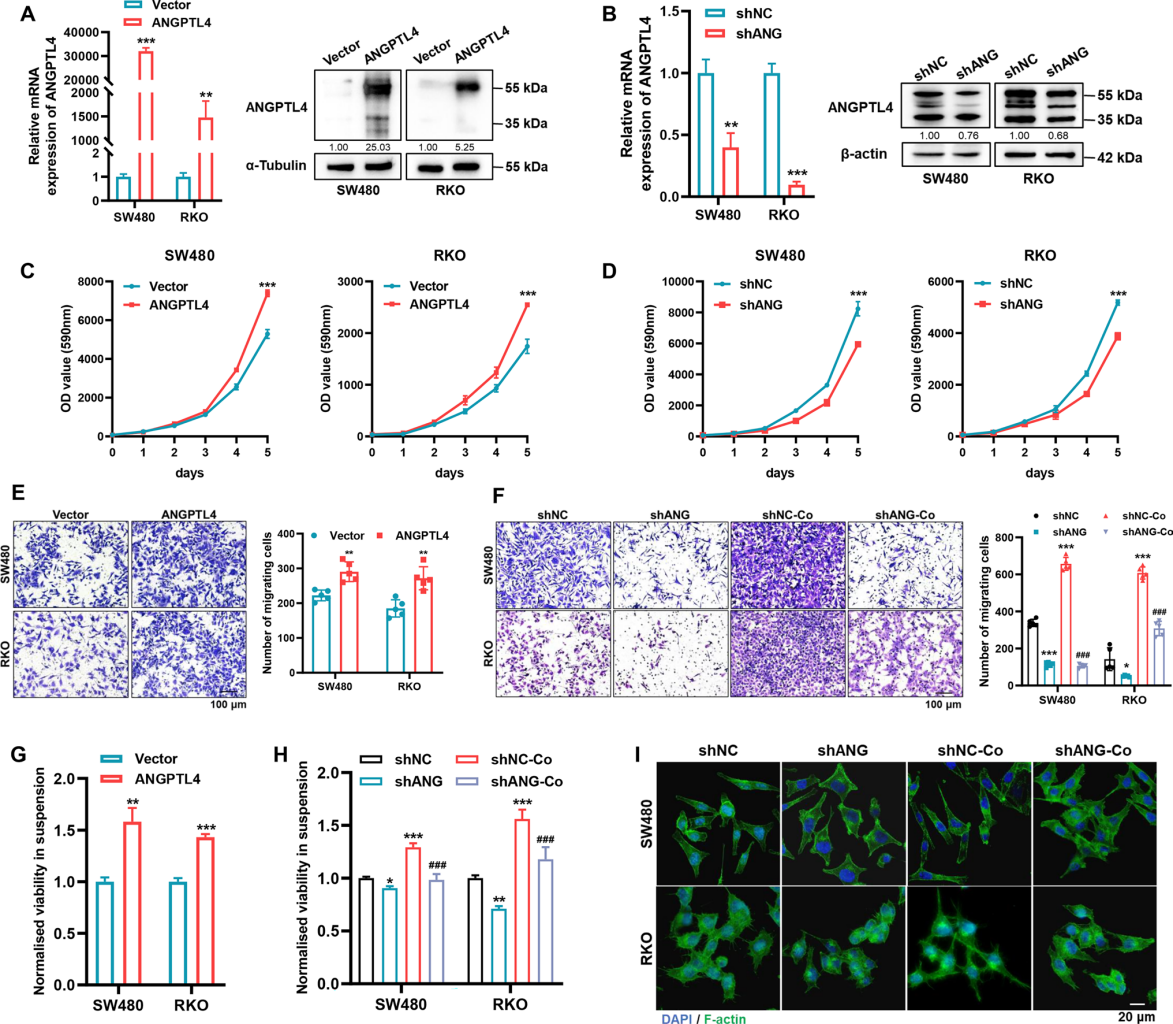
ANGPTL4 sustains migration capacity and anoikis resistance in CRC cells. **A**, **B** qRT-PCR and western blot analysis to detect ANGPTL4 expression level in SW480 and RKO cells transfected with ANGPTL4-overexpression plasmid or ANGPTL4-targeting shRNA. **C**, **D** Cell proliferation assay in the indicated CRC cells. **E**, **F** Transwell assay results showing that ANGPTL4 modulated the migration ability of CRC cells and ANGPTL4 blockade abolished the promotive effect induced by ADSCs. Scale bar 100 µm. **G**, **H** Anoikis resistance was assessed by measuring the viability of cells in suspension. *represents comparison with the shNC group and # represents comparison with the shNC-Co group in (**H**). **I** Phalloidin staining showing the morphologic changes and rearrangement of actin filaments in the indicated cells. Scale bar 20 µm. Data are shown as mean ± SD of at least three independent experiments in (**A**–**H**) (Student’s *t *test. *p < 0.05, **p < 0.01, ***p < 0.001, ^###^*p* < 0.001)

To elucidate the influence of ANGPTL4 on the behavior of CRC cells, we transfected SW480 and RKO cells with ANGPTL4-overexpression plasmid or ANGPTL4-targeting shRNA, which were validated by qRT-PCR and western blot (Fig. 4A, B; Supplementary Fig. S3A, B). The cell proliferation assay demonstrated that the exogenous expression of ANGPTL4 augmented the proliferation rate of CRC cells, and opposite results were observed in cells transfected with the ANGPTL4-targeting shRNA (Fig. 4C, D). In addition to accelerating growth, ANGPTL4 endowed CRC cells with metastasis potential coupled with anoikis resistance. Moreover, weakening the endogenous expression of ANGPTL4 counteracted the ADSCs-induced promotive effect (Fig. 4E–H). Phalloidin staining suggested that ANGPTL4 rearranged actin filaments, and CRC cells with ANGPTL4 blockade kept a round morphology even in the presence of ADSCs (Fig. 4I). The GSEA of data from TCGA-CRC and GSE17536 revealed that ANGPTL4 promoted the enrichment of genes contributing to ECM receptor interaction and focal adhesion (Supplementary Fig. S3C–F). These results posit ANGPTL4 as a vital gene that sustains metastasis potential and anoikis resistance in CRC cells.

### ADSCs endow CRC cells with migration ability via the TGF-β1/SMAD3/ANGPTL4 axis

**Fig. 5:**
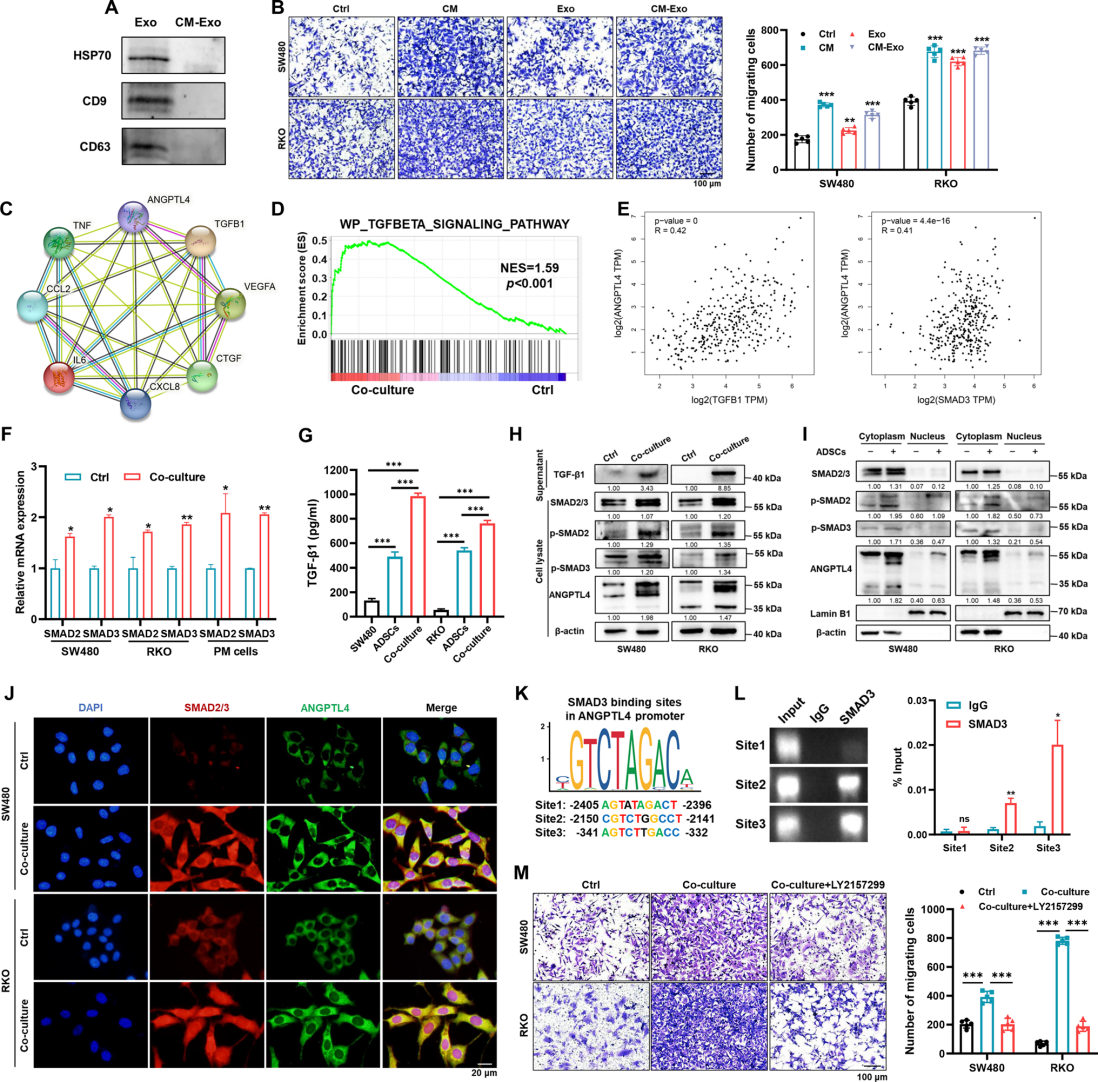
ADSCs endow CRC cells with a metastatic phenotype via the TGF-β1/SMAD3/ANGPTL4 axis. **A** Characterization of exosomes derived from ADSCs by western blot. **B** Migration assay of CRC cells treated with ADSCs-CM, ADSCs-exosomes, or ADSCs-CM depleted exosomes. Scale bar 100 µm. **C** PPI analysis between ANGPTL4 and cytokines abundantly secreted by ADSCs. **D** GSEA of the gene signature associated with the TGF-β signaling pathway in SW480 cells co-cultured with ADSCs. **E** Correlation of ANGPTL4 with TGFB1 and SMAD3 expression in CRC tissues from GEPIA2. **F** qRT-PCR analysis for SMAD2 and SMAD3 in indicated CRC cells. **G** ELISA assay for TGF-β1 in the supernatant of CRC cells, ADSCs and co-culture system. **H**, **I** Western blot analysis for key molecules of the TGF-β/SMAD signaling pathway in indicated cells. **J** Immunofluorescence staining showing the expression and localization of SMAD2/3 (red) and ANGPTL4 (green). Nuclei were counterstained with DAPI (blue). Scale bar 20 µm. **K** SMAD3 binding sites in the ANGPTL4 promoter, predicted using the JASPAR database. **L** ChIP assay performed on SW480 cells to confirm the SMAD3 binding sites in the ANGPTL4 promoter. **M** Migration assay in CRC cells treated as indicated. Cells were treated with or without 10 µM LY2157299 for 48 h. Scale bar 100 µm. Data are shown as mean ± SD of at least three independent experiments in (**B**, **F**, **G**, **L**, **M**) (Student’s *t *test. ns, not significant; *p < 0.05, **p < 0.01, ***p < 0.001)

Stromal cells usually modulate tumor cells through paracrine activity or the exosome [30]. To identify the critical factor through which ADSCs mediated the metastasis phenotype of CRC cells, we first isolated ADSCs-exosomes, which was identified by western blot analysis of exosome markers (Fig. 5A). Next, the transwell assay indicated that both ADSCs-exosomes and the ADSCs-CM depleted of exosomes increased the number of migrating CRC cells, and it was more remarkable with the latter treatment (Fig. 5B). Given this phenomenon, we hypothesized that ADSCs influenced the functions of CRC cells principally through paracrine factors. The cytokines in high abundance produced by mesenchymal stem cells include VEGF, IL-6, TGF-β1, CTGF, CXCL8, CCL2, TNF, and so on [9, 15]. Hence, we evaluated the correlation between these cytokines and ANGPTL4, the key manipulated gene in CRC cells affected by ADSCs, by performing a PPI analysis using the String database (https://cn.string-db.org/) (Fig. 5C). Previous reports indicated that TGF-β primed breast cancer for lung metastasis seeding and brain metastasis via ANGPTL4 [22, 31]. Furthermore, the GSEA of RNA sequencing data uncovered that the TGF-β signaling pathway was activated in SW480 cells co-cultured with ADSCs (normalized enrichment score (NES) = 1.59, *p* < 0.001) (Fig. 5D). Additionally, the expression level of ANGPTL4 was positively correlated with that of TGFB1 or SMAD3 in CRC tissues from GEPIA2 database (R = 0.42, *p* < 0.001 and R = 0.41, *p* < 0.001, respectively) (Fig. 5E).

Given that the exact mechanism of regulating ANGPTL4 by the TGF-β/SMAD signaling pathway is still unclear, we first analyzed the transcriptional levels and found that the SMAD2 and SMAD3 mRNA expression levels were markedly elevated in CRC cells in the presence of ADSCs (Fig. 5F). To investigate the source of TGF-β1, we performed the ELISA assay to detect the supernatant TGF-β1 of CRC cells, ADSCs and co-culture system. The TGF-β1 level in ADSCs supernatant was much higher than SW480 and RKO cells (3.7 and 9.7 times respectively) (Fig. 5G). Moreover, the western blot analysis suggested that TGF-β1 was much more abundant in the culture supernatant of co-cultured CRC cells with ADSCs than in that of CRC cells alone. Concomitantly, total SMAD2/3, p-SMAD2, p-SMAD3 and ANGPTL4 were upregulated by ADSCs (Fig. 5H, I). The immunofluorescence staining also showed a higher expression of total SMAD2/3 and more noticeable nuclear localization, coupled with a prominent activation of ANGPTL4 in SW480 and RKO cells co-cultured with ADSCs (Fig. 5J). Next, JASPAR database (https://jaspar.genereg.net/) was applied to identify the putative binding sites of the transcription factor SMAD3 in ANGPTL4 promoter located 3000 bp upstream of the transcription start site and three sites were acquired with a relative score of 80% or over (Fig. 5K). The subsequent ChIP assay confirmed that the ANGPTL4 promoter region exhibited a significant enrichment in two SMAD-binding sites after immunoprecipitation with the anti-SMAD3 antibody (Fig. 5L).

To ascertain whether targeting the TGF-β/SMAD signaling pathway could prevent the migration of CRC cells, LY2157299 (also known as Galunisertib), a robust inhibitor of TGF-β receptor, was applied to further experiments. Importantly, blockading the TGF-β signaling notably hampered the migration ability and almost restored the basal number of migrating CRC cells in the presence of ADSCs (Fig. 5M). Altogether, these findings posit the TGF-β1/SMAD3/ANGPTL4 axis as a dominant pathway through which ADSCs endow CRC cells with the metastatic phenotype.

### ADSCs activate glycolysis via the TGF-β1/SMAD3/ANGPTL4 axis in CRC cells

**Fig. 6:**
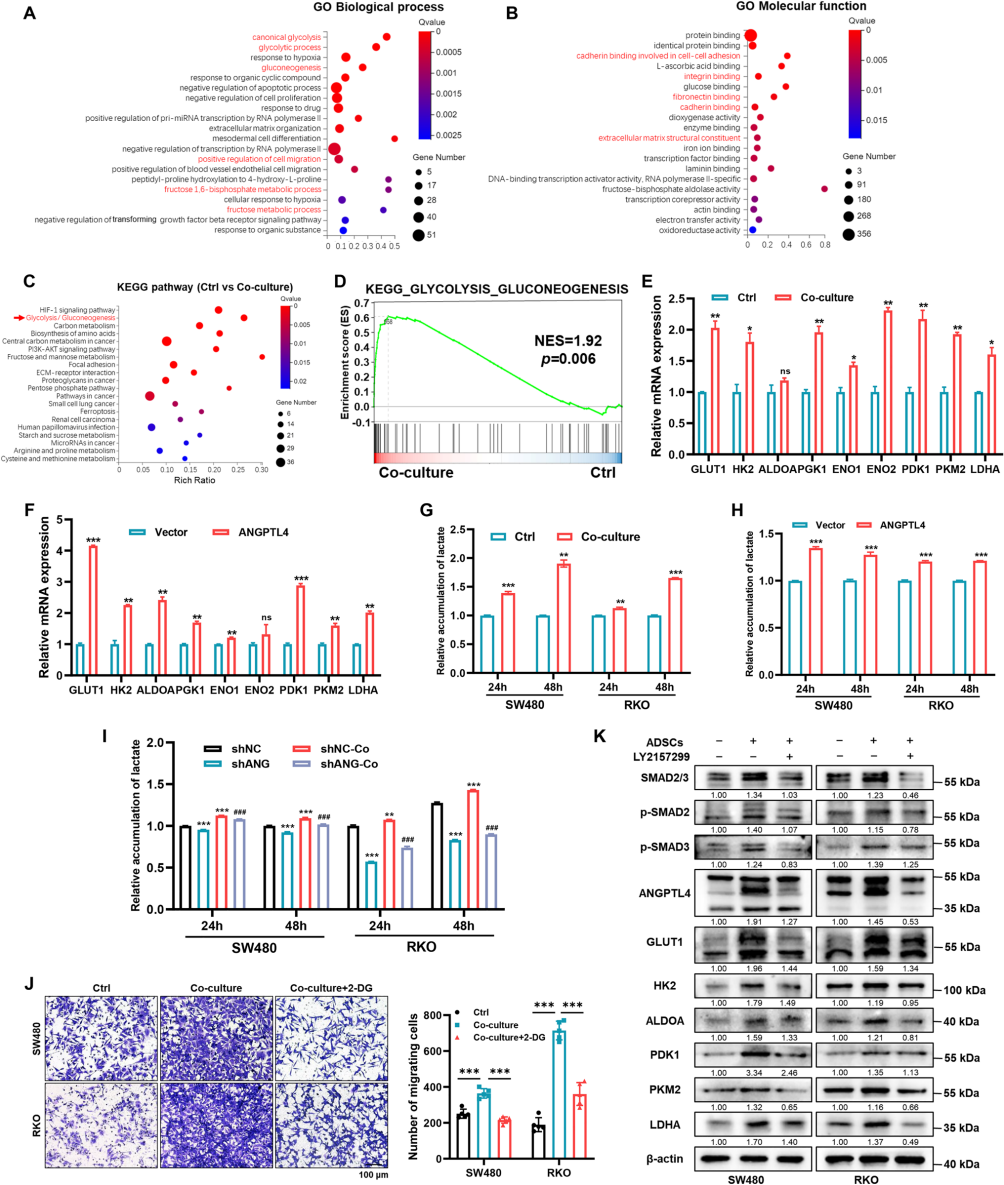
ADSCs endow CRC cells with activated glycolysis via the TGF-β1/SMAD3/ANGPTL4 axis. **A**, **B** GO enrichment analysis (biologic process and molecular function) of DEGs identified in SW480 cells cultured with or without ADSCs. **C** KEGG pathway enrichment analysis of DEGs among SW480 cells cultured with or without ADSCs. **D** GSEA of the gene signature associated with glycolysis and gluconeogenesis in SW480 cells co-cultured with ADSCs. **E**, **F** qRT-PCR analysis of the key metabolic enzymes participating in glucose metabolism.** G**–**I** Quantification of lactate production in CRC cells treated as indicated. *Represents comparison with the shNC group and # represents comparison with the shNC-Co group in (**I**). **J** Migration assay in CRC cells treated as indicated. Cells were treated with or without 10 mM 2-DG for 48 h. Scale bar 100 µm. **K** Western blot analysis results suggesting that treatment with 10 µM LY2157299 for 48 h to inhibit TGF-β/SMAD signaling in CRC cells remarkably reduced the expression of key metabolic enzymes involved in glycolysis. Data are shown as mean ± SD of at least three independent experiments in (**E**–**J**) (Student’s *t *test. ns, not significant; *p < 0.05, **p < 0.01, ***p < 0.001, ^###^*p* < 0.001)

As stated above, ADSCs endow CRC cells with high expression of ANGPTL4, which contributes to modulating glycolysis in tumors [19]. Consistently, the pathway enrichment analysis of DEGs in RNA sequencing highlighted an enrichment in glycolysis and gluconeogenesis (Fig. 6A–C), which was then confirmed by GSEA (NES = 1.92, *p* = 0.006) (Fig. 6D). Moreover, the qRT-PCR analysis indicated that the key metabolic enzymes participating in glucose metabolism were elevated in SW480 cells co-cultured with ADSCs or transfected with ANGPTL4-overexpression plasmid (Fig. 6E, F). The accumulation of lactate is a hallmark of activated glycolysis [32]. Additionally, both ADSCs and the exogenous ANGPTL4 expression accelerated lactate accumulation in CRC cells, whereas ANGPTL4 knockdown restored the lactate in the culture supernatant to basal levels (Fig. 6G–I). Furthermore, treatment with 2-Deoxy-D-glucose (2-DG), a glucose analog acting as a competitive inhibitor on glucose metabolism, inhibited the ADSC-induced migration of CRC cells (Fig. 6J). Finally, to determine whether blocking TGF-β/SMAD signaling could inhibit glycolysis, we quantified the expression of key metabolic enzymes in CRC cells treated with 10 µM LY2157299 for 48 h. The results suggested that blocking TGF-β/SMAD signaling with LY2157299 remarkably reduced the expression of key metabolic enzymes involved in glycolysis (Fig. 6K).

Based on these results, we concluded that ADSCs induce active glycolysis through the TGF-β1/SMAD3/ANGPTL4 axis, ultimately enhancing the migration capacity of CRC cells.

### Blocking the TGF-β1/SMAD3/ANGPTL4 axis hampers ADSCs-induced intraperitoneal dissemination of CRC cells in vivo

**Fig. 7:**
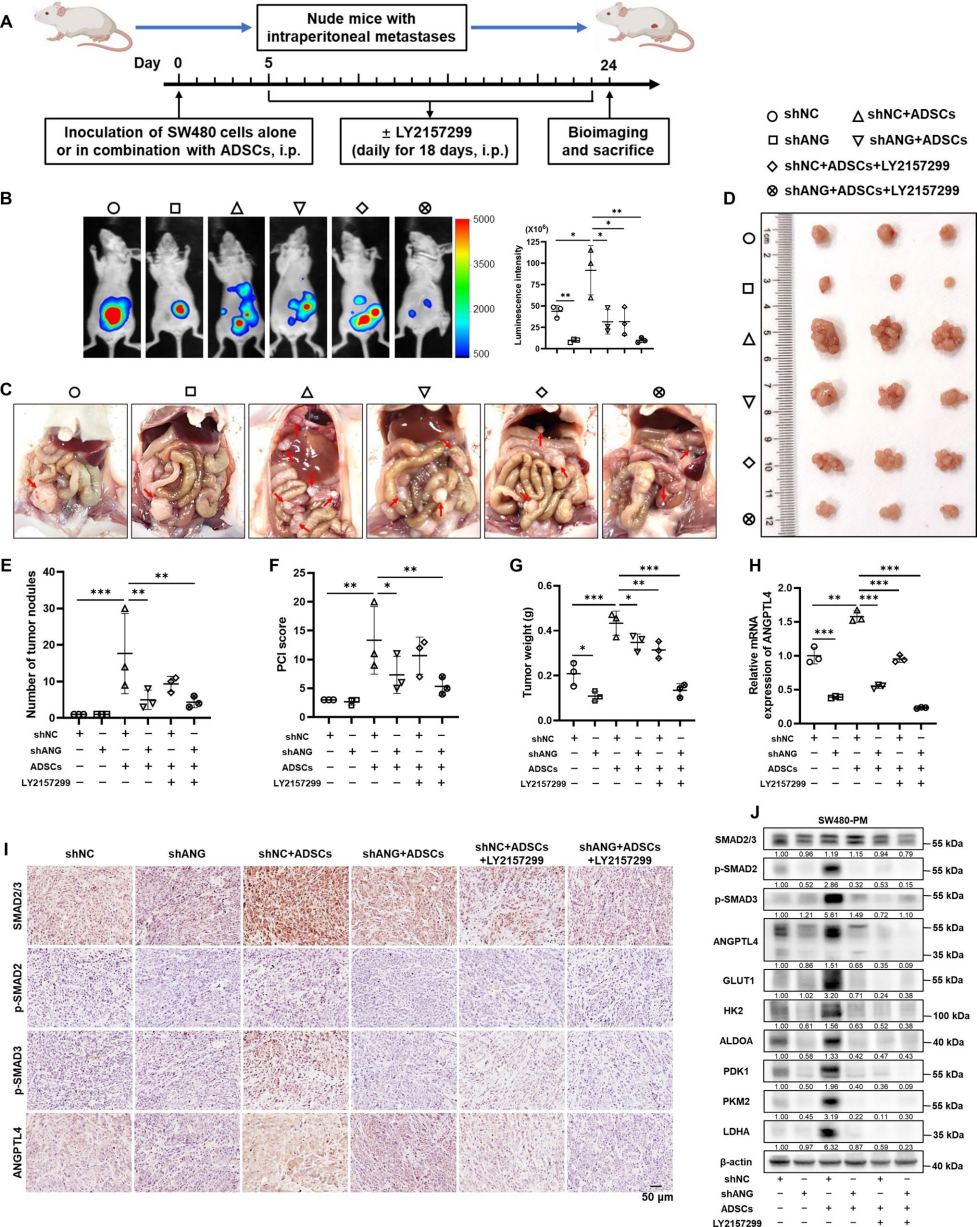
Dual-targeting of TGF-β signaling and ANGPTL4 strikingly impede the ADSCs-induced intraperitoneal dissemination of CRC cells.** A** Schematic diagram of CRC intraperitoneal dissemination models showing time points of treatment in mice. **B** In vivo bioluminescence showing the intraperitoneal tumors in mice treated as indicated. **C**, **D** The mice were sacrificed and all the intraperitoneal tumors were collected. **E**–**G** The number of tumor nodules, PCI score and tumor weight in mice treated as indicated (n = 3). **H** qRT-PCR analysis of ANGPTL4 in tumors. **I** Representative images of IHC staining for tumors dissected from mice with corresponding treatments. **J** Western blot analysis for key molecules involved in TGF-β signaling and glycolysis. Data are shown as mean ± SD in (**E**–**H**) (Student’s *t *test. *p < 0.05, **p < 0.01, ***p < 0.001)

To further investigate whether disturbing the TGF-β1/SMAD3/ANGPTL4 axis induced by ADSCs could impair the peritoneal seeding capacity of CRC cells in vivo, SW480 cells alone or co-injected with ADSCs were inoculated in the peritoneal cavity of mice. Five days later, the mice received an intraperitoneal injection of 20 mg/kg of LY2157299 every day for 18 days (Fig. 7A). Subsequently, bioimaging and anatomy analysis revealed that blocking TGF-β signaling and knocking down ANGPTL4 strikingly impeded the ADSCs-induced intraperitoneal metastasis formation by SW480 cells in vivo (Fig. 7B–D). Accordingly, the number of tumor nodules, PCI score and tumor weight were lower in mice with combined treatment than those with a single therapeutic regimen (Fig. 7E–G). The expression levels of SMAD2, SMAD3 and ANGPTL4 were assessed by qRT-PCR and IHC staining in intraperitoneal tumors (Fig. 7H, I). Importantly, the dual-target treatment abolished the ADSCs-induced upregulation of glucose metabolic enzymes in tumors (Fig. 7J).

**Fig. 8:**
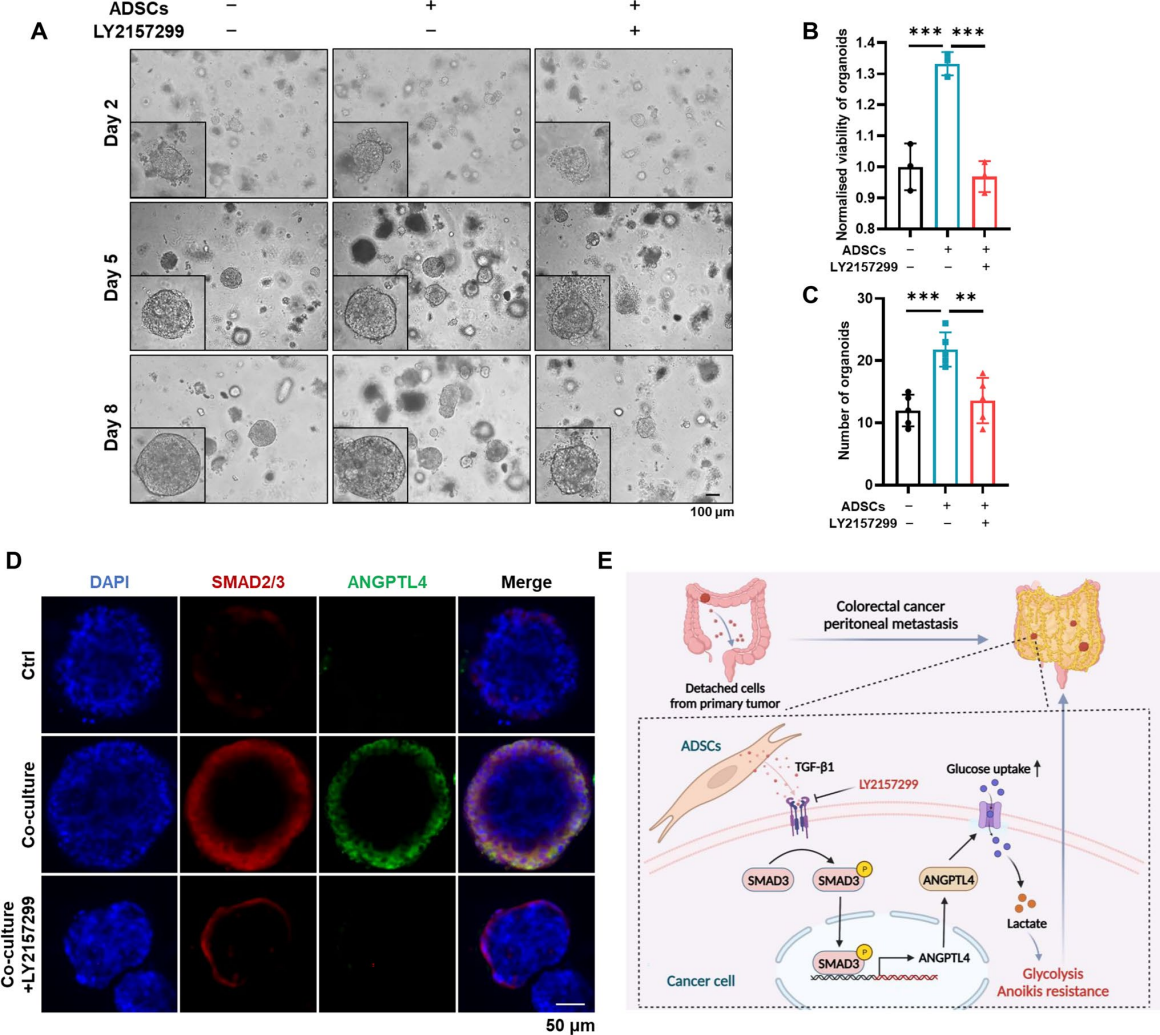
Targeting TGF-β signaling suppresses the growth of patient-derived organoids sustained by ADSCs. **A** Phase-contrast images of CRC organoids at 2, 5 and 8 days. Scale bar 100 µm. **B**, **C** The viability and number of CRC organoids treated as indicated. **D** Immunofluorescence staining of SMAD2/3 (red) and ANGPTL4 (green) in CRC organoids. Nuclei were counterstained with DAPI (blue). Scale bar 50 µm. **E** Schematic illustration showing that ADSCs make CRC cells acquire activated glycolysis and anoikis resistance via the TGF-β1/SMAD3/ANGPTL4 axis, ultimately facilitating peritoneal metastasis and cancer progression. Data are shown as mean ± SD of at least three independent experiments in (**B**, **C**) (Student’s *t *test. **p < 0.01, ***p < 0.001)

Patient-derived organoid is proposed as a promising preclinical model thanks to the high similarity of molecular characteristics and heterogeneity with original tumor tissue [24, 33]. Thus, CRC tissues were isolated to generate organoids, which was subsequently dosed with LY2157299 to block TGF-β signaling. The results indicated that ADSCs prominently accelerated the growth of organoids, whereas LY2157299 reduced the ADSCs-induced promotion of viability and increase in the number of organoids (Fig. 8A–C). In line with the results in SW480 and RKO cells (Fig. 5I), ADSCs increased the expression of SMAD2/3 and ANGPTL4 in organoids, whereas LY2157299 inhibited this increase (Fig. 8D).

These results suggest that ADSCs, via the TGF-β1/SMAD3/ANGPTL4 axis, promote glycolysis and increase intraperitoneal dissemination in CRC cells in vivo (Fig. 8E). Moreover, blocking TGF-β signaling and knocking down ANGPTL4 prevent ADSCs from inducing the aggressive phenotype of CRC cells.

## Discussion

Distant metastasis formation is a complex multistep process and the crosstalk between tumor cells and supporting cells in the tumor microenvironment has been an enduring subject in cancer progression. The widely accepted “seed and soil” hypothesis states that the metastasis cascade depends on the mutual communication between the initiating tumor cells (“the seeds”) and the target organ microenvironment (“the soil”) [34, 35]. Numerous chemokines secreted by tumor cells recruit various stromal cells and establish a specific microenvironment that enables initiating tumor cells to colonize and germinate in metastatic sites [7, 36]. The adipose-rich omentum possesses various types of stromal cells, including mesenchymal stem cells, fibroblasts, adipocytes and macrophages, which tend to be remodeled and recruited by malignant tumor cells [7, 13]. Ovarian cancer has an intrinsic metastatic tropism for the adipose-rich omentum [13, 37]. The visceral ADSCs promote intraperitoneal dissemination by the increasing expression of the chemokines IL-6, MIP-2, and MCP-1 in ovarian cancer [10]. In addition, lipids derived from cancer-associated fibroblasts promote CRC peritoneal metastasis by increasing cell membrane fluidity [38]. These observations suggest that the peritoneal ecosystem acts as a key driver of CRC peritoneal metastasis and hinders treatments.

ADSCs recruited to the tumor environment have gained increasing attention in recent years, as they drive tumor progression by interacting with tumor cells in multiple cancers, including ovarian cancer, breast cancer, endometrial cancer, as well as CRC [7, 39–41]. High levels of VEGF, FGF and SDF1-α secreted by omental ADSCs enhance the vascularization and survival of endometrial tumor [42]. In addition, omental ADSCs promote glycolysis, reduce oxidative stress and induce chemotherapy resistance by supporting nitric oxide homeostasis through paracrine metabolite secretion in ovarian and endometrial cancer cells [39]. The crosstalk between tumor cells and ADSCs also determines the invasive phenotype of CRC cells, as tumor-released neurotrophins recruit ADSCs within the tumor mass and ADSCs-derived secretory factors sustain the stemness of tumor cells [9]. Notably, bioengineered ADSCs with targeting ability have been utilized as a precise tumor treatment. Chemotherapy combinated with ADSCs genetically engineered to express human carboxylesterase-2, can overcome drug resistance and exhibit survival benefits in ovarian cancer with intraperitoneal metastasis [43]. In this study, we first isolated ADSCs from visceral adipose tissues of CRC patients and verified their multi-lineage differentiation potential. In accordance with previous finding [44], data from online databases indicated that ADSCs infiltration was increased in CRC peritoneal metastases and associated with a poor prognosis in CRC patients. Furthermore, we used an animal model to investigate whether ADSCs in the peritoneal microenvironment affect CRC peritoneal metastasis and confirmed that co-injecting ADSCs with SW480 cells facilitated intraperitoneal seeding.

ANGPTL4 may be a key regulator in complex signaling pathways and enhance the malignancy of cancer cells through diverse functions [16]. It’s reported that ANGPTL4 facilitates the colonization of cancer cells to new tissues by increasing vascular permeability [22, 45]. Additionally, ANGPTL4 mediates tumor proliferation and metastasis through metabolic pathways in both dyslipidemia-associated CRC and *F. nucleatum*-related CRC [19, 20]. ANGPTL4 also contributes to EMT-mediated chemotherapy resistance by empowering cancer cells with metabolic flexibility [21]. Herein, we posit that ANGPTL4 is an independent prognostic factor negatively associated with the survival of CRC patients and that ADSCs-induced ANGPTL4 expression endows CRC cells with invasive potential.

The activation of TGF-β signaling is perceived as a characteristic of CRC-related peritoneal metastatic disease [46]. Accumulating evidence reveals that blocking the cross-talk between cancer cells and the microenvironment using TGF-β signaling inhibitors prevents immune evasion and metastasis in CRC [47–49]. A recent study revealed that the trafficking and secretion of ANGPTL4 via vesicle-associated actin assembly mediated by TGF-β is important for cancer development [50]. In addition, TGF-β signaling primes breast tumors for lung metastasis seeding and brain metastasis by inducing ANGPTL4 expression [22, 31]. In the present study, we found that ADSCs-induced TGF-β/SMAD signaling enhanced the growth and migration of CRC cells by transcriptionally regulating ANGPTL4. Moreover, LY2157299, a CRC treatment currently undergoing clinical trials [51, 52], reverses the promotive action of ADSCs by targeting the TGF-β receptor in vitro and in vivo. The phenomenon was also confirmed by patient-derived organoid model, a promising tool for fundamental research and translational medicine, as it reserves the molecular characteristics of original tumor and subtly simulates the tumor microenvironment of the human body [24, 33, 53]. The application of the organoid model makes the results more reliable and confirms that LY2157299 is a promising drug candidate.

Deregulating cellular metabolism is one of the hallmarks of cancer [54]. The Warburg effect in cancer cells prompts the accumulation of diverse metabolites, including lactate, lipids and amino acids, in tumor microenvironment [32]. Thereinto, the accumulation of lactate facilitates the survival and immune evasion of tumor cells [55, 56]. In gastric cancer, intraperitoneal seeding is accompanied by enhanced glycolysis mediated by stem cell factor SALL4, which transcriptionally activates HK2 expression [57]. Shed cancer cells from primary tumor acquire anoikis resistance through a cascade of metabolic adaptation mediated by glycolytic enzymes [58, 59]. Resistance to anoikis promotes anchorage-independent survival for detached cancer cells in the peritoneal cavity, which is a critical event in the peritoneal metastasis cascade [28, 59, 60]. Our results manifested that ADSCs activate glycolysis and induce anoikis resistance by upregulating the expression of ANGPTL4 in CRC cells.

## Conclusion

This study shows that tumor-infiltrating ADSCs prime CRC for peritoneal metastasis by promoting glycolysis and anoikis resistance. Mechanistically, the ADSCs-activated TGF-β1/SMAD3 signaling pathway transcriptionally upregulates the expression of ANGPTL4, which endows tumor cells with an invasive phenotype and is associated with a poor prognosis in CRC patients. Importantly, a treatment targeting the TGF-β1/SMAD3/ANGPTL4 axis effectively prevented the intraperitoneal seeding of CRC cells in vivo. Thus, a combined approach targeting TGF-β signaling through LY2157299 and inhibiting endogenous ANGPTL4 expression would be a sensible therapeutic strategy for CRC peritoneal metastasis.

### Supplementary Information

Below is the link to the electronic supplementary material.

Supplementary file1 (DOCX 25 KB)

Supplementary file2 (PNG 552 KB)

Supplementary file3 (PNG 1432 KB)

Supplementary file4 (PNG 1010 KB)

## Data Availability

All the data that support the findings of this study are available from the corresponding authors upon reasonable request.

## Abbreviations

CRC: Colorectal cancer
ADSCs: Adipose-derived stem cells
ANGPTL4: Angiopoietin Like 4
qRT-PCR: Quantitative real-time PCR
CM: Conditioned medium
PM: Peritoneal metastasis
ChIP: Chromatin immunoprecipitation
TCGA: The Cancer Genome Atlas
GEO: Gene Expression Omnibus
GSEA: Gene set enrichment analysis
PPI: Protein–protein interaction
IHC: Immunohistochemistry
EMT: Epithelial-mesenchymal transition
DEGs: Differentially expressed genes
NES: Normalized enrichment score
