# Supplementary material for: Adipose-derived stem cells promote glycolysis and peritoneal metastasis via TGF-β1/SMAD3/ANGPTL4 axis in colorectal cancer
Source: Cell Mol Life Sci. 2024 Apr 21;81(1):189. doi: 10.1007/s00018-024-05215-1 (PMC11033247; doi:10.1007/s00018-024-05215-1)
Supplement: Supplementary file 1 — Supplementary file1 (DOCX 25 KB) [file 18_2024_5215_MOESM1_ESM.docx]

**Supplementary Methods**

**Cell migration and invasion assay**

The 24-well plate was filled with DMEM, conditioned medium or ADSCs suspension containing 10% FBS, and then 5×10^4^ SW480 cells or 1×10^5^ RKO cells were suspended in serum-free medium and placed into an 8.0-µm pore polycarbonate membrane insert with or without diluted Matrigel (1:40). After 48 h of incubation, the cells were fixed and stained. The cells on the lower surface were counted after wipe of the cells on the upper surface by swabs.

**Cell proliferation assay and wound healing assay**

For cell proliferation assay, SW480 and RKO cells were seeded in 96-well plates at a density of 1×10^3^ cells per well and treated as indicated. The viability of cells was detected every day by resazurin (Meilunbio, Dalian, China).

For wound healing assay, SW480 and RKO cells were seeded into 12-well plates at a density of 6×10^5^ per well. When the confluency reached to 80%, straight lines were scratched by a 0.1-10 µl pipette tip and images were taken by microscope.

**Anoikis assay**

A total of 1×10^5^ SW480 or RKO cells were suspended in 1 ml medium and transferred into a well of 24-well ultra-low attachment plate (Corning) to induce anoikis. The cells were treated as indicated for 48 h. To measure the viability of CRC cells in suspension, the cells were incubated with 100 µl resazurin solution per well for 1 h, and the optical density (OD) value was detected at the wavelength of 590 nm using SpectraMax M4 Multimode microplate readers (Molecular Devices, San Jose, United States).

**RNA isolation and PCR**

Total RNA was extracted from CRC tissues and cells with Trizol solution (TaKaRa, Japan). Hifair III 1st Strand cDNA Synthesis SuperMix (Yeasen, Shanghai, China) and SYBR Green Primix *Pro Taq* HS (Accurate Biology, Changsha, China) were used in reverse transcription PCR and qRT-PCR following the manufacturer’s instructions. Gene expression of interest was normalized to ACTB, and data were analyzed with the

2^–ΔΔCt^ method. The primers used in this study are listed in the Supplementary Table S2.

**Western blot and immunohistochemistry (IHC) staining**

RIPA Lysis Buffer and Minute™ Cytoplasmic and Nuclear Extraction Kits for Cells (SC-003, Invent Biotechnologies, Beijing, China) were used to extract total protein or cytoplasmic and nuclear protein according to the manufacturer’s protocols. Protein samples were separated by 10% SDS-PAGE and transferred onto PVDF membranes, which were then incubated with primary antibodies at 4 °C overnight and with HRP conjugated secondary antibodies (Beyotime, Shanghai, China) for 1 h at room temperature. Protein bands were visualized by an automatic chemiluminescence image analysis system (Tanon, Shanghai, China). The primary antibodies used for western blot in this study are listed in the Supplementary Table S3.

IHC staining was performed according to the instruction for IHC kit (PV-6001, ZSGB-BIO, Beijing, China). The primary antibodies used for IHC in this study are listed in the Supplementary Table S3.

**Immunofluorescence staining**

The cells on slides were fixed and with 4% paraformaldehyde, permeabilized with 0.5% Triton X-100 and blocked with 2% BSA blocking solution. Then, the cells were incubated with primary antibodies at 4 °C overnight and IgG-Alexa Fluor 594 antibody (1:100, ZF-0516, ZSGB-BIO) and IgG-Alexa Fluor 488 (1:100, ZF-0512, ZSGB-BIO) for 60 min in the dark. Last, the cells were counterstained with DAPI (P0131, Beyotime) and imaged by Olympus fluorescence microscope. The primary antibodies used for immunofluorescence staining in this study are listed in the Supplementary Table S3.

For staining actin filaments (F-actin), Phalloidin-iFluor 488 Reagent (1:500, ab176753, abcam) was utilized according to protocol.

**Plasmid, siRNA, lentiviral construction and cell transfection**

The coding sequence of ANGPTL4 (NM_139314.3) was cloned into the expression vector pENTER (WZ Biosciences, Jinan, China) to construct ANGPTL4-overexpressing plasmid and cells were transfected with Lipofectamine™ 3000 Transfection Reagent (Invitrogen, Carlsbad, USA) according to the manufacturer's instructions. Three siRNA sequences (GenePharma, Shanghai, China) were constructed to interfere the endogenous expression of ANGPTL4, then si_ANG_3 was chosen to construct the lentivirus-delivered shRNA (Genechem, Shanghai, China) for ANGPTL4 knockdown. The ANGPTL4-targeting siRNA sequences are listed in the Supplementary Table S2.

| **Supplementary Table 1. Clinical characteristics of CRC patients for the sources of ADSCs** | | | | | | |
| --- | --- | --- | --- | --- | --- | --- |
| **Sequence number** | **Gender** | **Age** | **Body Mass Index** | **Tumor differentiation** | **TNM Staging** | **AJCC Staging** |
| P01 | Female | 61 | 25.6 | Moderate | T4N2M1 | IV |
| P02 | Male | 49 | 25.1 | / | / | / |
| P03 | Female | 62 | 20.4 | Poor | T4N0M0 | II |
| P04 | Female | 48 | 31.2 | Moderate | T3N0M0 | II |
| P05 | Female | 74 | 23 | Moderate | T3N0M0 | II |
| P06 | Female | 57 | 25.4 | Moderate | T3N1M0 | III |
| P07 | Female | 22 | 18.8 | Moderate | T1N0M0 | I |
| P08 | Female | 58 | 19.1 | Poor | T3N2M1 | IV |
| P09 | Male | 60 | 16.5 | Moderate | T4N0M0 | II |
| P10 | Male | 67 | 16.9 | Well | T3N2M0 | III |

**Supplementary Table S2.** Primers and siRNA used in this study

| Gene | Forward (5' to 3') | Reverse (5' to 3') |
| --- | --- | --- |
| ACTB | CATGTACGTTGCTATCCAGGC | CTCCTTAATGTCACGCACGAT |
| ANGPTL4 | GTCCACCGACCTCCCGTTA | CCTCATGGTCTAGGTGCTTGT |
| SMAD2 | TTCAGTTCCGCCTCCAATCG | GCAAGCCACGCTAGGAAAAC |
| SMAD3 | TCTCCCCGAATCCGATGTCC | GCTGGTTCAGCTCGTAGTAGG |
| GLUT1 | CAGAAGGTGATCGAGGAGTTC | AGAGAAGGAGCCAATCATGCC |
| HK2 | GAGCCACCACTCACCCTACT | CCAGGCATTCGGCAATGTG |
| ALDOA | ATGCCCTACCAATATCCAGCA | GCTCCCAGTGGACTCATCTG |
| PGK1 | TGGACGTTAAAGGGAAGCGG | GCTCATAAGGACTACCGACTTGG |
| ENO1 | GCCGTGAACGAGAAGTCCTG | ACGCCTGAAGAGACTCGGT |
| ENO2 | CCGGGAACTCAGACCTCATC | CTCTGCACCTAGTCGCATGG |
| PDK1 | CTGTGATACGGATCAGAAACCG | TCCACCAAACAATAAAGAGTGCT |
| PKM2 | ATGTCGAAGCCCCATAGTGAA | TGGGTGGTGAATCAATGTCCA |
| LDHA | ATGGCAACTCTAAAGGATCAGC | CCAACCCCAACAACTGTAATCT |
| ChIP-Site1 | GCGAGACTCCTTCTCAAA | GTCCCACAGCACAAAACA |
| ChIP-Site2 | AGGAGGAGCTTGCATTCTG | AGGCTGCCACTCATACACTT |
| ChIP-Site3 | GGTTCAAGCGATTCTACTGC | GTGGGAGACGTGACTCTTCTTT |
| siRNA | **Sense (5' to 3')** | **Antisense (5' to 3')** |
| Negative control | UUCUCCGAACGUGUCACGUTT | ACGUGACACGUUCGGAGAATT |
| si_ANG_1 | GGGACGAGAUGAAUGUCCUTT | AGGACAUUCAUCUCGUCCCTT |
| si_ANG_2 | GCAGGAUCCAGCAACUCUUTT | AAGAGUUGCUGGAUCCUGCTT |
| si_ANG_3 | CCACAAGCACCUAGACCAUTT | AUGGUCUAGGUGCUUGUGGTT |

**Supplementary Table S3.** Primary antibodies used in this study

| **Antibodies** | **Source** | **Identifier** | **Usage** |
| --- | --- | --- | --- |
| CD73 | Proteintech | 12231-1-AP | 1:500 for IF |
| CD90 | Proteintech | 66766-1-Ig | 1:250 for IF |
| CD105 | Proteintech | 65048-1-Ig | 1:200 for IF |
| EpCAM | Proteintech | 66316-1-Ig | 1:200 for IF |
| α-Tubulin | Rayantibody | RM 2007 | 1:10000 for WB |
| β-actin | CST | #8457 | 1:1000 for WB |
| ANGPTL4 | abcam | ab206420 | 1:1000 for WB  1:2000 for IHC |
| ANGPTL4 | Proteintech | 67577-1-Ig | 1:250 for IF |
| N-cadherin | Proteintech | 22018-1-AP | 1:4000 for WB  1:2000 for IHC |
| E-cadherin | Proteintech | 60335-1-Ig | 1:4000 for WB  1:4000 for IHC |
| Vimentin | Proteintech | 60330-1-Ig | 1:4000 for WB  1:8000 for IHC |
| Snail | Proteintech | 13099-1-AP | 1:1000 for WB |
| Slug | Proteintech | 12129-1-AP | 1:1000 for WB |
| HSP70 | CST | #4876 | 1:1000 for WB |
| CD9 | CST | #13174 | 1:1000 for WB |
| CD63 | CST | #52090 | 1:1000 for WB |
| TGF-β1 | Proteintech | 21898-1-AP | 1:2000 for WB |
| SMAD2/3 | CST | #8685 | 1:1000 for WB  1:1000 for IHC  1;500 for IF |
| SMAD3 | CST | #9523 | 1:100 for ChIP |
| p-SMAD2 | CST | #3108 | 1:1000 for WB  1:500 for IHC |
| p-SMAD3 | CST | #9520 | 1:1000 for WB  1:500 for IHC |
| Lamin B1 | Proteintech | 66095-1-Ig | 1:5000 for WB |
| GLUT1 | CST | #12939 | 1:1000 for WB |
| HK2 | CST | #2867 | 1:1000 for WB |
| ALDOA | CST | #8060 | 1:1000 for WB |
| PDK1 | CST | #13037 | 1:1000 for WB |
| PKM2 | CST | #4053 | 1:1000 for WB |
| LDHA | CST | #3582 | 1:1000 for WB |

**Supplementary Figure Legends**

**Supplementary Figure S1 Survival analysis of ADSCs markers in CRC patients from PROGgeneV2 database.** **(A-F)** Overall survival plots of CRC patients in GSE17537 and GSE16125 stratified by gene expression level of NT5E, THY1, and ENG, respectively.

**Supplementary Figure S2 ANGPTL4 is upregulated in CRC cells co-cultured with ADSCs and is a risk factor for CRC death. (A)** Heatmap of the top 50 feature genes. **(B)** Heatmap of hierarchical clustering showing genes differentially expressed in SW480 cells cultured with or without ADSCs. **(C)** qRT-PCR analysis of ANGPTL4 mRNA in cells co-cultured with ADSCs. **(D-F)** Expression level of ANGPTL4 along with progression of clinical stage. **(G, H)** Overall survival curve of CRC patients from GSE29621 and GSE39582 divided by ANGPTL4 expression. **(I)** Multivariate Cox regression analysis concerning clinical features and ANGPTL4 expression level (data from GSE17536). Student’s *t*-test for (C, D, F) (ns, not significant; * *p* < 0.05, ** *p* < 0.01). One-way ANOVA for (E).

**Supplementary Figure S3 Verification of transfection efficiency and GSEA by expression level of ANGPTL4. (A, B)** qRT-PCR and western blot analysis were performed to detect ANGPTL4 expression level in CRC cells transfected with ANGPTL4-targeting siRNA and the sequence of si_ANG_3 was subsequently applied to construct ANGPTL4-targeting shRNA**. (C-F)** GSEA revealed that ANGPTL4 promoted the enrichment of genes contributing to ECM receptor interaction and focal adhesion (data from TCGA-CRC and GSE17536). Data are shown as mean ± SD in (A) (Student’s *t*-test. *** *p* < 0.001).
