# Supplementary figures and images for: Adipose-derived stem cells promote glycolysis and peritoneal metastasis via TGF-β1/SMAD3/ANGPTL4 axis in colorectal cancer
Source: Cell Mol Life Sci. 2024 Apr 21;81(1):189. doi: 10.1007/s00018-024-05215-1 (PMC11033247; doi:10.1007/s00018-024-05215-1)

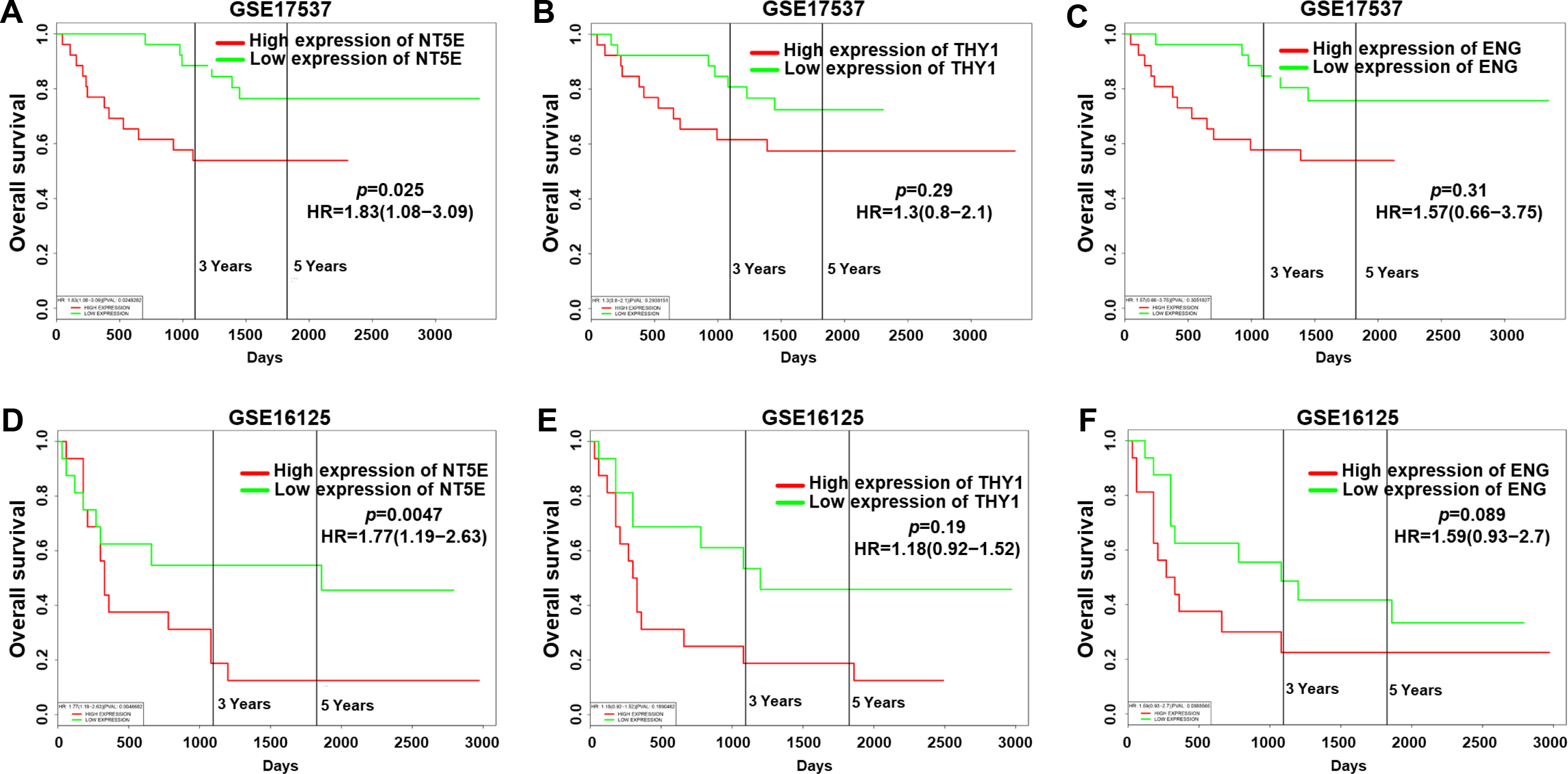

Supplement: Supplementary file 2 — Supplementary file2 (PNG 552 KB) [file 18_2024_5215_MOESM2_ESM.png]

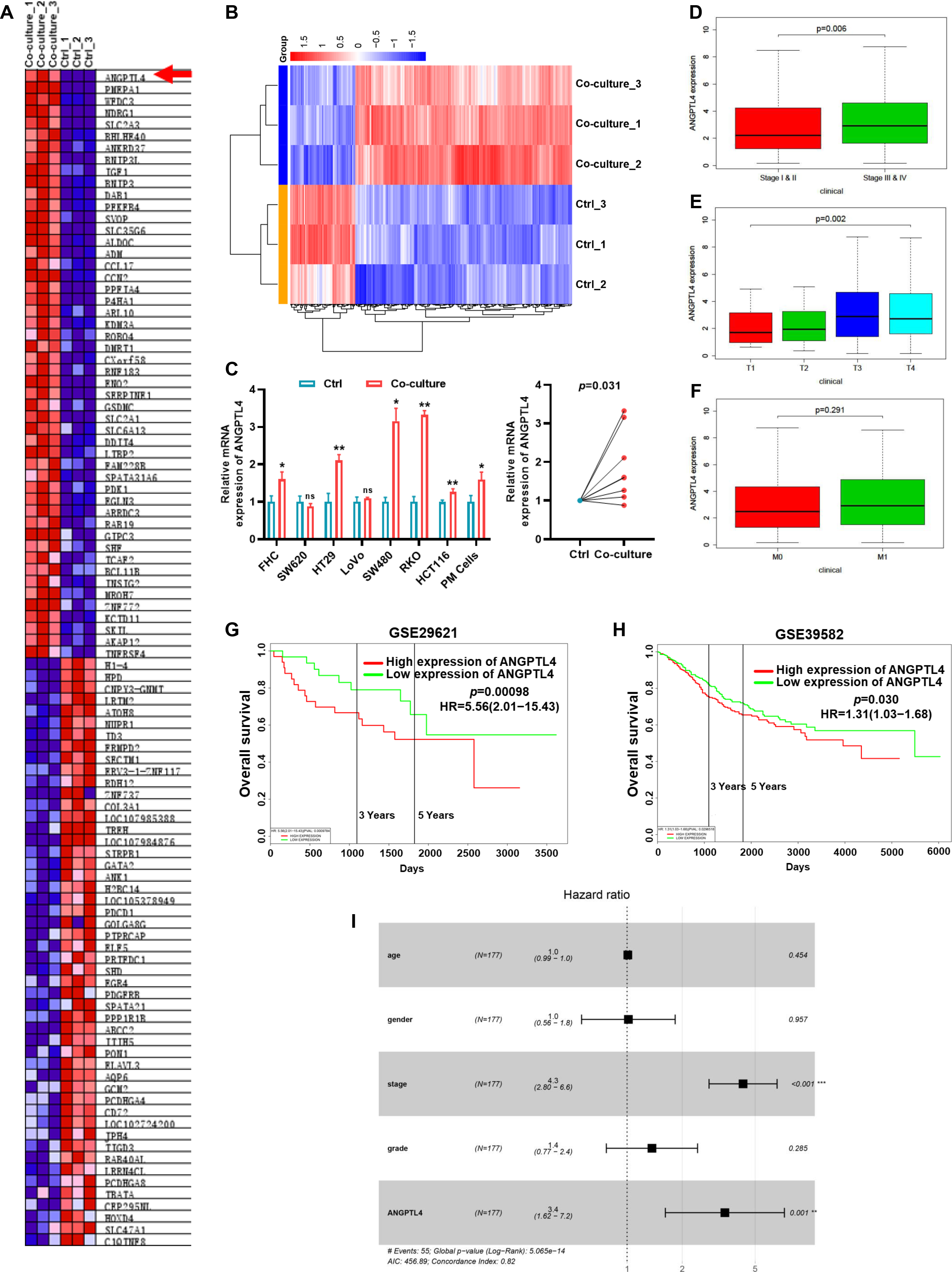

Supplement: Supplementary file 3 — Supplementary file3 (PNG 1432 KB) [file 18_2024_5215_MOESM3_ESM.png]

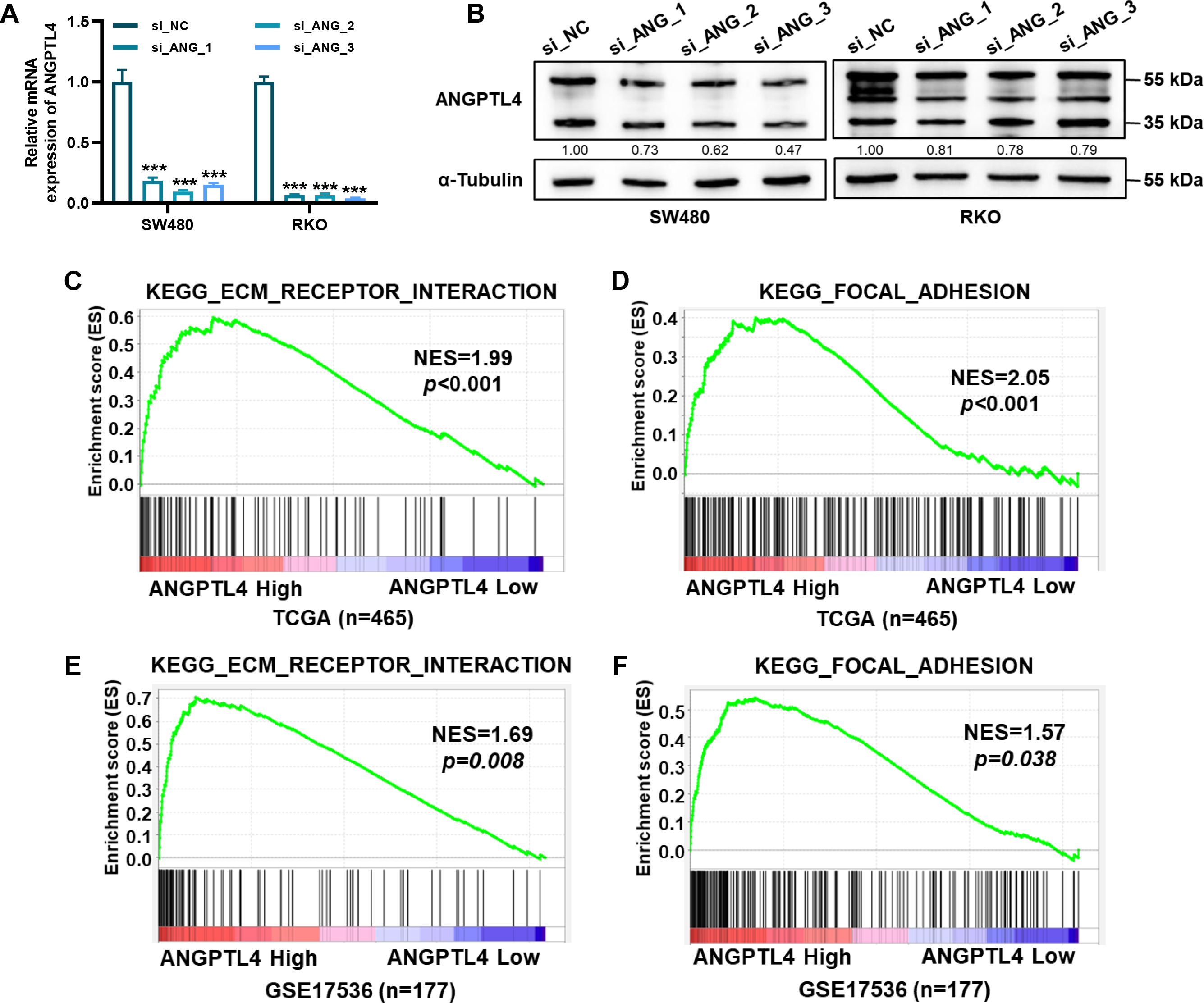

Supplement: Supplementary file 4 — Supplementary file4 (PNG 1010 KB) [file 18_2024_5215_MOESM4_ESM.png]
